# Restoring p53 in cancer: the promises and the challenges

**DOI:** 10.1093/jmcb/mjz063

**Published:** 2019-08-22

**Authors:** Guillermina Lozano

**Affiliations:** Department of Genetics, University of Texas MD Anderson Cancer Center, Houston, TX 77030, USA

## A tribute to Dr Arnold J. Levine

This perspective was written in celebration of Dr Arnold J. Levine’s 80th birthday. I chose to write this prospective about restoration of p53 activity in tumors for two reasons: Arnie (as we all know him) has become interested in restoring p53 activity in tumor cells and such a review did not exist. Arnie was also my postdoctoral advisor and writing this perspective is just a small tribute for the huge impact he has had on my research career (and that of many others), from supporting me when I left his laboratory for a faculty position before I had even published a paper, to serving as a sounding board for numerous ideas. Arnie is an amazing thinker. He is able to almost effortlessly incorporate new knowledge into current thinking and collate a big picture. It has been my pleasure to interact with him scientifically on numerous occasions. I want to thank Arnie from the bottom of my heart for sharing with me his love of science, his critical thinking skills, and his friendship.

## p53 pathway inactivation

The p53 tumor suppressor is inactivated in most (and possibly all) cancers via various mechanisms indicating a potent role in inhibiting tumor cell growth (Wasylishen and Lozano, 2016). The idea of restoring wild-type p53 function in tumors has gained traction and is likely feasible in some tumors. However, since the pathway is inactivated by deletion or mutation of p53 or by overexpression of its inhibitors Mdm2 and Mdm4, amongst other mechanisms, p53 reactivation will have to be uniquely tailored to the genetics of a specific tumor type. For example, tumors with elevated levels of the p53 inhibitors Mdm2 or Mdm4, which retain a wild-type *p53* gene, should be treated with drugs that disrupt binding of these inhibitors to p53 to restore p53 function. Tumors lacking p53, on the other hand, need to reintroduce p53 through a virus encoding wild-type p53 or convert mutant p53 to wild type. Genetically modified mouse models have been used to examine p53 restoration in various contexts and will be reviewed here. Tumor responses have been heterogeneous suggesting that other factors contribute to the outcome. Resistance mechanisms will also likely emerge and need to be understood in more detail.

## Restoring p53 in tumors lacking p53

Initial p53 reactivation studies in mouse models employed *p53* alleles that were not functional but could be reactivated in a Cre-dependent manner. The Jacks laboratory generated a wild-type *p53* locus with a lox-stop-lox (LSL) cassette in the first intron effectively eliminating p53 expression but allowing p53 re-expression in the presence of Cre recombinase (Ventura et al., 2007). They also generated a *Cre-ER^T2^* mouse in which the Cre recombinase is active only in the presence of Tamoxifen. Similar to germline *p53^−/−^* mice, the *LSL-p53* homozygous mice with the *Cre-ER^T2^* allele develop autochthonous lymphomas and sarcomas due to loss of p53 function. Tamoxifen injections allow tumors to re-express p53 and thus can be used to study p53 restoration. A total of 70% (7/10) of these tumors regressed and 20% showed tumor stasis (1 T-cell lymphoma and 1 osteosarcoma). One tumor lost the *Cre-ER^T2^* allele and thus grew like controls. In this context, p53 restoration led to apoptosis in lymphomas but decreased proliferation, cell cycle arrest, and senescence in sarcomas (Table 1). In these experiments, all tumors with restoration of wild-type p53 responded albeit to varying depth.

**Table 1:** p53 restoration models.

**Genotype**	**p53 loss/restoration**	**Tumors**	**Effects on tumor growth**	**Cellular response**	**Reference**
*p53^LSL/LSL^*;*Cre-ER^T2^*	Germline	Lymphomas	Regression	Apoptosis	Ventura et al. (2007)
		Sarcomas	Regression; stasis	Cell cycle arrest; senescence	
*p53^Neo/−^*;*CAG-CreER*	Germline	Lymphomas	Regression	Apoptosis	Wang et al. (2011)
		Angiosarcomas	Regression	Senescence	
*p53^Neo/R172H^*;*CAG-CreER*	Germline	Lymphomas	Stasis	Apoptosis; senescence	Wang et al. (2011)
		Angiosarcomas	Stasis	Senescence; decreased cell proliferation	
*p53^Neo/R172H^*;*Cre-ER^T2^*	Germline	Lymphomas	Mixed	Apoptosis	Larsson et al. (2018)
*HaRas^V12^*;*p53* shRNA	Carcinoma only	Hepatocellular carcinomas	Regression	Senescence; immune response	Xue et al. (2007)
*Eμ-myc*;*p53ER^TAM/+^*	B-cells	B-cell lymphomas	Delay	Apoptosis	Martins et al. (2006)
*Mdm2Tg*;*p53^Neo/Neo^*;*CAG-CreER*	Germline	Angiosarcomas	Stasis	Decreased proliferation; senescence	Li et al. (2014)

**Figure 1:**
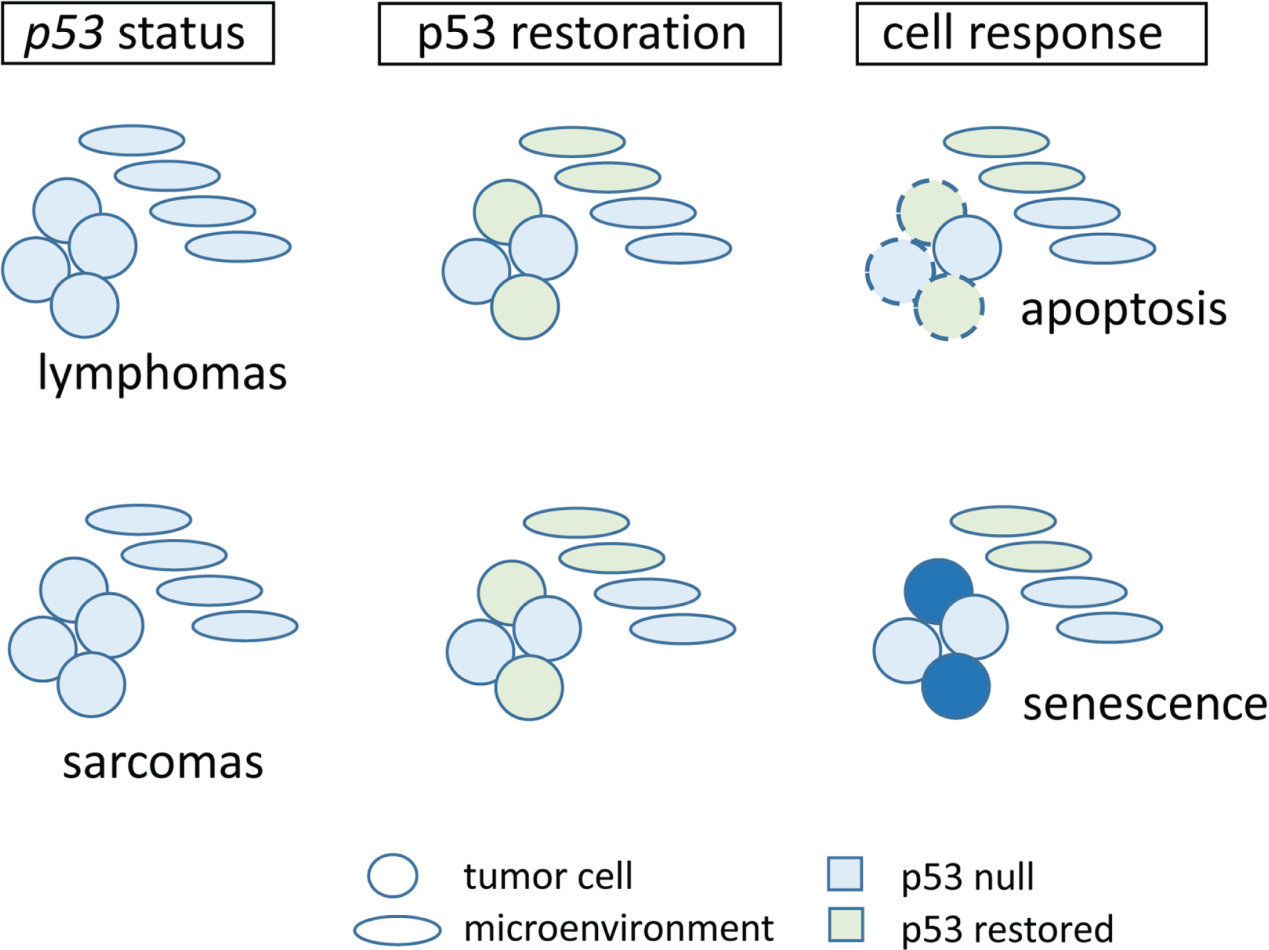
Tumors lacking p53 show varied responses to p53 restoration. Tumor cells are circles; cells of the TME are depicted as ovals. Light blue cells have lost p53, and green cells have restored p53.

In another study, Wang et al. (2011) used a *p53* allele with insertion of a *Neo* cassette (*p53^Neo^*) that dramatically reduces expression of p53 to  ~7% of wild type. The p53 response upon restoration in autochthonous tumors that develop due to *p53* loss was similar with lymphomas inducing apoptosis and angiosarcomas inducing senescence. Tumors regressed and in some cases completely disappeared, which coincided with increased survival of the mice. In both of the above studies, it should be noted that the *p53* alleles are germline such that all cells of the mouse have less p53 than normal, and Cre recombination restores p53 not just in tumor cells (Figure 1). Importantly, even though restoration of p53 by Cre recombinase does not occur in every tumor cell, tumor regression was observed, suggesting that p53 restoration in a few cells is sufficient to exert a cytotoxic effect on neighboring cells. This bystander effect had been observed in humans treated with a p53 adenovirus (Roth, 2006; Waku et al., 2000). A more in-depth understanding of this phenomenon is needed.

## Restoring p53 in tumors with p53 missense mutations

**Figure 2:**
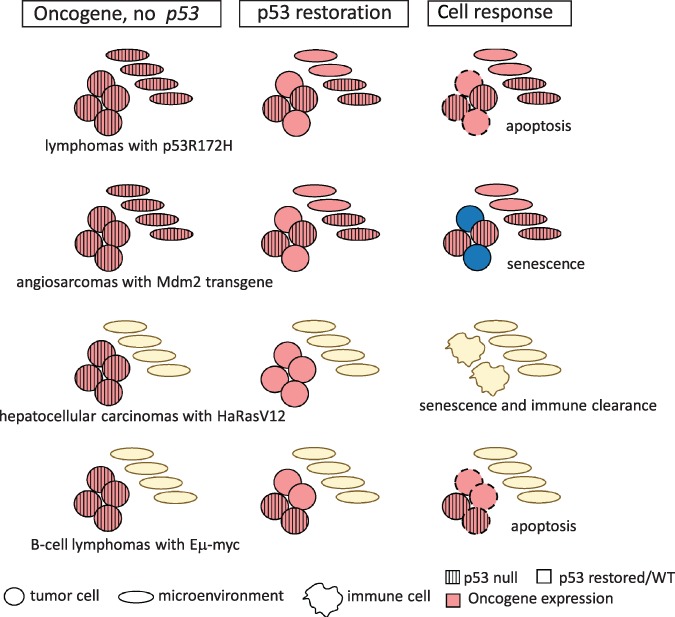
Response to p53 restoration in tumors with *p53* loss and a cooperating oncogene. Tumor cells are circles; cells of the TME are depicted as ovals. Pink cells have an oncogenic mutation, and striped cells depict *p53* loss; yellow cells are normal; blue cells are senescent, and dotted outlines depict apoptotic cells.

The majority of *p53* mutations that occur in human cancers are missense mutations (Hainaut and Pfeifer, 2016). p53 missense mutants are known to have gain-of-function (GOF) activities that usurp normal transcriptional programs (Brosh and Rotter, 2009; Kim and Lozano, 2018) and dominant-negative effects on wild-type p53 (Goh et al., 2011). As thus, it was important to evaluate the response of restoring p53 activity in tumors with *p53* missense mutations. Therefore, the restoration of p53 was also evaluated in tumors with the p53R172H hotspot mutation. Mice with a p53R172H germline mutation develop tumors that are highly metastatic as compared to *p53* heterozygous mice (Lang et al., 2004; Olive et al., 2004). Mice containing one *p53^R172H^* allele, one *p53^Neo^* allele (that expresses very low levels of p53 that can be restored with Cre recombination), and the *CAG-CreER* Tamoxifen-inducible transgene develop lymphomas and sarcomas. p53 was restored with the addition of Tamoxifen and in contrast to *p53*-null tumors that regressed in the two studies described above, lymphomas and sarcomas in this case showed tumor stasis but not tumor regression (Table 1). Lymphomas with restored p53 had both apoptotic and a few senescent cells, while angiosarcomas had senescent cells (Figure 2). The levels of restored wild-type p53 are likely competing with mutant p53. Additionally, though these tumors initiated with a *p53* missense mutation with GOF activities, the tumors may have evolved differently, compared to tumors lacking *p53*, adding to the diminished response.

The response to p53 restoration is, in fact, very heterogeneous. A similar genetic model with p53R172H and *p53^Neo^* but using the *Rosa26-Cre-ER^T2^* knock-in allele identified a range of responses (Larsson et al., 2018). Of 24 lymphomas examined with restored p53, 50% responded (i.e. tumors regressed), 37% did not respond, and 12% were stable. The responding lymphomas died by apoptosis. RNA sequence comparisons of responders and non-responders identified activation of the tumor necrosis factor (TNF) pathway in responders. Fas ligand (FasL), a member of the TNF family, was one of the most significantly upregulated genes. IPA analysis implicated RARγ as a pharmacological target upstream of FasL and it is activated by retinoic acid. Treatment with a synthetic retinoid in a syngeneic transplant model was additive with p53 restoration to delay tumor progression and increase survival. In fact, this retinoid also boosted the response of sensitive tumors, further implicating this pathway in the response to p53 restoration.

This study emphasizes our lack of understanding of the cellular response as it shows that restoring p53 to similar levels in 24 lymphomas produces a wide range of responses. These data support the heterogeneity observed in human cancers in response to various drugs and afford a model in which the molecular events can be dissected to understand the factors that lead to non-responsiveness.

## Restoring p53 in tumors driven by oncogenes

Oncogenes such as *Ras* and *Myc* are also drivers of tumorigenesis. Tumors with these alterations often also have *p53* mutations. To determine whether reintroduction of p53 might have an effect in these kinds of cancers, embryonic liver progenitor cells containing retroviruses expressing HRasV12, a tetracycline transactivator protein, and a tet-responsive *p53* shRNA were seeded into the livers of athymic nude mice (Xue et al., 2007). Invasive hepatocarcinomas develop in these mice due to the cooperation of HRasV12 and *p53* loss. Doxycycline treatment shuts off the tet-responsive *p53* shRNA restoring p53 activity. p53 restoration had a dramatic effect on tumor growth. Complete regression of liver carcinomas occurred even if p53 was restored for only 4 days (Figure 2). Restoration did not induce apoptosis but did cause decrease in proliferation and a senescent phenotype (Table 1). Further analyses showed that p53 restoration induced activation of cytokines by tumor cells and transcripts for macrophages, neutrophils, and natural killer cells. Inhibition of these cell types with drugs or neutralizing antibodies slowed tumor regression, indicating that the immune response was important in tumor clearance. The immune response in this case may have overruled the senescent phenotype. It is important to note that in these experiments, doxycycline-mediated suppression of the p53 shRNA occurs in the majority, if not all tumor cells, which may account for the complete response.

The effects of p53 restoration were also examined in a B-cell lymphoma model driven by *Eμ-myc*. In this case, Martins et al. (2006) used a switchable *p53* allele called *p53ER^TAM^*. This makes a p53 fusion protein with little activity and as such contributes to tumor development. All tested B-cell lymphomas from *Eμ-myc*;*p53ER^TAM/+^* mice lost the remaining wild-type *p53* allele either by loss of heterozygosity or point mutations. Addition of Tamoxifen restored p53 activity, caused massive apoptosis, and increased survival of mice (Figure 2).

In this model, resistance to p53 restoration was also examined and arose through one of two mechanisms: loss of *p53ER^TAM^* allele, rendering Tamoxifen injections useless, or loss of *Arf*. *Arf* encodes a short protein that interacts with Mdm2 and disrupts its inhibition of p53 activity. In cells, *Arf* loss leads to increased binding of Mdm2 to p53 and thus dampened p53 activity. Understanding tumor resistance is critical to predicting successful combination therapies that will be long lasting.

*Mdm2* is also an oncogene as it is amplified or overexpressed in many cancers and displays mutual exclusivity with p53 alterations (Wasylishen and Lozano, 2016). To determine the efficacy of restoring p53 in this context, a tumor prone *Mdm2* transgenic mouse with low levels of p53 (due to a germline *p53^Neo^* allele) was used (Li et al., 2014). Restoration of p53 in angiosarcomas in ~30% of tumor cells suppressed tumor growth and prolonged survival. p53 restoration inhibited proliferation in a sustained manner, as at end point (1 month), none of the Tamoxifen-treated Mdm2 transgenic mice had died, while 80% of the untreated mice with tumors did. While this was a proof-of-principle study in which restoration of p53 in Mdm2-overexpressing angiosarcomas shows efficacy, the *p53* genotype was such that p53 was restored in both Cre-expressing tumor and normal cells. Thus, intrinsic vs. extrinsic effects on tumor suppression could not be distinguished in this model.

## Common themes in therapeutic restoration of p53

Thus, restoration of p53 has therapeutic efficacy although the mechanisms varied dependent on tumor type (Table 1). The most common response to p53 restoration in tumors is not apoptosis but instead results in decreased proliferation and senescence. Lymphomas tend to induce apoptosis, while on the other hand sarcomas and carcinomas induce senescence. While the cell cycle arrest and senescent functions of p53 are also tumor suppressive (Liu et al., 2004; Chen et al., 2005), they do not actually eradicate the tumor cell. An important question that has yet to be addressed is whether the p53-induced cell cycle arrest/senescent responses can be turned into an apoptotic one. While senescence was first described in tissue culture, it is difficult to study *in vivo*. Moreover, senescence may not be a dead-end *in vivo*. Senescence is known to produce senescence-associated secretory proteins (SASP), which include cytokines and chemokines with the potential to fuel tumor cell growth (Rao and Jackson, 2016). At least in one breast cancer study in mice, a p53-induced senescence response to doxorubicin treatment actually was worse for tumor progression in comparison to tumors lacking *p53* (Jackson et al., 2012). Thus, the triggers that convert cell cycle arrest to cell death (via whatever mechanism) need to be understood and explored in more detail. Understanding the tissue-specific nature of p53 target gene activation and subsequent responses is essential.

The caveats of the above studies are many: the studies were performed in mice not humans; most studies involved germline *p53* alterations such that the stroma and immune environment are mutant for p53 (except in the liver model); genetic reconstitution of p53 is permanent and does not mimic drug pharmacology. However, even a partial response appears to be effective in slowing tumor growth. In most of these studies as well, the tumor microenvironment (TME) has *p53* alterations, distorting interpretation of the data. We also need to expand these studies beyond the tumor types (lymphomas and sarcomas) that occur with germline loss of *p53*.

## Clinical studies to restore p53 activity with small molecules

Several classes of Mdm2 inhibitors (Mdm2i) have been developed to restore p53 activity (recently reviewed by Wang et al., 2017). These inhibitors prevent Mdm2 from binding and inhibiting p53 functions. Several of these inhibitors have progressed to the clinic with important observations. First, Mdm2i are toxic to hematopoiesis resulting in thrombocytopenia and neutropenia. This was predicted from various mouse studies in which decreased Mdm2 level below 50% leads to increased p53 activity, defects in hematopoiesis, and bone marrow failure (Pant et al., 2012, 2016). Second, treatment with Mdm2i resulted in the emergence of p53 mutations (Wang et al., 2017). This last observation suggests that mechanisms for restoring p53 will stress tumor cells to delete *p53*. This was observed in the Jacks study above (Ventura et al., 2007) where loss of *Cre-ER^T2^* occurred in one tumor and in the Martins et al. (2006) study where one mechanism of resistance to *Eμ-myc*;*p53ER^TAM/+^*-induced B-cell lymphomas was loss of *p53ER^TAM^*. This selective pressure further supports the notion that all cancers must perturb the p53 pathway in order to initiate tumor growth.

Other small molecules to reactivate p53 are aimed at converting mutant p53 to wild-type p53. Several of these, MIRA, RITA, and Prima1, were identified as p53-reactivating drugs, although current knowledge suggests that some have p53-independent activities (Cheok and Lane, 2017). How much of the effect is due to p53 reactivation and to what extent p53 has to be restored in specific tumor cells are important remaining questions. Also, it is clear from numerous studies that not all p53 mutants are the same (Freed-Pastor and Prives, 2012). Thus, some of these drugs may work better with some p53 mutants than others.

## Beyond restoring p53 function, tumors are addicted to mutant p53

In addition to the observations that mutant p53 proteins exhibit GOF activities, a growing body of evidence suggests that cells may be addicted to mutant p53 expression. Experiments using siRNA knockdown of mutant p53 in cancer cell lines showed a higher apoptotic response to drug treatment (Bossi et al., 2006). Mutant *p53* depletion experiments show decreases in cell growth rate, viability, replication, and clonogenicity. Constitutive inhibition of mutant p53 reduced tumor growth in nude mice and showed reduced stromal invasion and angiogenesis (Bossi et al., 2008). Prives and colleagues showed that mutant p53 depletion in breast cancer cells (MDA-MB-231 cells with p53R280K and MDA-MB-468 with p53R273H) in 3D culture leads to phenotypic reversion to more normal, differentiated structures with hollow lumens (Freed-Pastor et al., 2012).

*In vivo* experiments show that mutant p53 ablation in spontaneously arising lymphomas and colorectal cancers curbs tumor growth (Alexandrova et al., 2015; Schulz-Heddergott et al., 2018). However, these studies were performed in mice with a germline floxed mutant *p53* allele resulting in stochastic mutant *p53* depletion in tumors, the TME, and immune system. Thus, in these experiments, some tumor cells and some cells of the TME and immune system retain the mutant *p53* allele, confounding the interpretation of these results. The immune system is relevant as, for example, it contributed to complete tumor regression in hepatocellular tumors discussed above (Xue et al., 2007). While these models demonstrate that tumors that develop with p53 missense mutations can become addicted to the mutant proteins and establish mutant p53 function as a viable therapeutic target, more clinically relevant models with somatic mutation of p53 in the context of a wild-type immune system and TME are essential to advance robust pre-clinical evaluation (Zhang et al., 2018).

In summary, restoring p53 has been analyzed via multiple systems in multiple tumor types. In the majority of cases, tumors slowed down and the mice lived longer although they still succumbed to the disease. This may be due to the fact that p53 was not restored in all tumor cells, except in hepatocarcinomas that completely regressed (Xue et al., 2007) or that other mechanisms dampen p53 restoration (high Mdm2 or mutant p53 with dominant-negative GOF activities). Importantly, researchers in the field still cannot predict what the p53 response will be: arrest, senescence, or apoptosis. This is an important question as ultimately, tumor cells need to be eliminated for the best response. A combinatorial use of p53 restoration with drugs that push cells into apoptosis or that unleash the immune system is essential for best results. Also, a better understanding of the tissue-specific nature of p53 targets that are activated deserves more attention, as these might provide alternative therapeutic targets.

*[I would like to thank Sydney Moyer, Amanda Wasylishen, and Shunbin Xiong for helpful comments. G.L. was supported by a grant from the National Institutes of Health (CA82577).]*
